# Carbon Footprint of Mediterranean Pasture-Based Native Beef: Effects of Agronomic Practices and Pasture Management under Different Climate Change Scenarios

**DOI:** 10.3390/ani10030415

**Published:** 2020-03-02

**Authors:** Giampiero Grossi, Andrea Vitali, Nicola Lacetera, Pier Paolo Danieli, Umberto Bernabucci, Alessandro Nardone

**Affiliations:** Department of Agricultural and Forestry Sciences (DAFNE), University of Tuscia, Viterbo 01100, Italy; g.grossi@unitus.it (G.G.); danieli@unitus.it (P.P.D.); bernab@unitus.it (U.B.); nardone@unitus.it (A.N.)

**Keywords:** greenhouse gases, soil management, mitigation, N_2_O, SOC, DNDC model, RCP

## Abstract

**Simple Summary:**

The livestock sector requires a significant amount of natural resources and has an important role in climate change. Although the carbon footprint has become a widely accepted indicator for assessing the greenhouse gases emitted per unit of product, due to the lack of a commonly accepted methodology, there are still few studies that have included soil organic carbon sequestration in their calculations. In this study, by including soil organic carbon dynamics, the carbon footprint of a Mediterranean pasture-based beef cattle farm was estimated using current weather data and farming management policies. Subsequently, different soil management strategies, grazing systems, and climate scenarios were compared to the current ones to investigate the effects of these variables on the greenhouse gases emitted. The results showed that the current beef carbon footprint could be significantly reduced by switching to reduced tillage systems. The modeled combination of no-tillage practices with higher organic fertilizer application rates showed a greater potential carbon footprint reduction. No significant differences were found between carbon footprint values modeled under different climate scenarios and grazing systems. By including a process-based model into its carbon footprint calculations, this study highlights the climate mitigation potential of different farming practices and the importance of considering soil carbon sequestration.

**Abstract:**

A better understanding of soil organic carbon (SOC) dynamics is needed when assessing the carbon footprint (CFP) of livestock products and the effectiveness of possible agriculture mitigation strategies. This study aimed (i) to perform a cradle-to-gate CFP of pasture-based beef cattle in a Mediterranean agropastoral system (ii) and to assess the effects on the CFP of alternative tillage, fertilizing, and grazing practices under current (NCC) and future climate change (CC) scenarios. Minimum (Mt) and no-tillage (Nt) practices were compared to current tillage (Ct); a 50% increase (Hf) and decrease (Lf) in fertilization was evaluated against the current (Cf) rate; and rotational grazing (Rg) was evaluated versus the current continuous grazing (Cg) system. The denitrification–decomposition (DNDC) model was run using NCC as well as representative concentration pathways to investigate the effects of farm management practices coupled with future CC scenarios on SOC dynamics, N_2_O fluxes, and crop yield. Within NCC and CtCf, an emission intensity of 26.9 ± 0.7 kg CO_2eq_ per kg live body weight was estimated. Compared to Ct, the adoption of Mt and Nt reduced the CFP by 20% and 35%, respectively, while NtHf reduced it by 40%. Conservation tillage practices were thus shown to be effective in mitigating greenhouse gas emissions.

## 1. Introduction

Greenhouse gas (GHG) emissions from the livestock sector amount to 14.5% of global anthropogenic emissions [1], and this number is expected to grow as a consequence of the increased demand for livestock products from developing countries [2]. To meet the future needs of the expanding human population, an increased efficiency of animal production systems coupled with a decrease in GHG emission intensity per unit of product must be targeted [3].

When assessing the environmental burden associated with livestock products, soil organic carbon (SOC) represents a large carbon pool sink that should be considered when evaluating the sustainability of agricultural systems [4].

In recent years, the promotion of less-intensive tillage practices [5], the use of organic fertilizers [6], and the adoption of rotational grazing systems [7] have been credited for mitigating climate change (CC) due to their positive effects on SOC preservation. 

Various methods have been used to estimate GHG emissions and sinks in agriculture carbon footprint (CFP) studies, ranging from a simple Tier 1 approach [8] to complex process-based models (Tier 3) capable of simulating carbon and nitrogen cycles [9]. On the one hand, the Tier 1 approach is still the most commonly used approach in the agriculture sector [10], but on the other hand, by considering the interactions between (i) climate, (ii) soil, and (iii) tillage practices, process-based models have been shown to be useful tools in simulating the long-term effects that these interactions have on crop yields, SOC dynamics, and GHG emissions [11]. Currently, the adoption of more accurate methods for the estimation of land-based emissions is recommended to improve the accuracy of CFP results [10]. 

Process-based models are based on biogeochemistry [12]: among them, the denitrification–decomposition (DNDC) model [13] has been applied in more than 30 countries across the world [14] and has been validated globally in over 100 studies, demonstrating its high accuracy [15]. 

Despite the fact that SOC sequestration in agricultural practices is a highly debated topic [16], investigating C sinks within agriculture CFP studies still requires further research to develop a common, reliable, and robust method [17]. As a result, there have been few CFPs that have included soil C dynamics in their results [18]. When SOC sequestrations have been included in beef cattle CFP studies [19,20], whole-farm GHG emissions have been found to decrease by 5%–43%. These findings underline the importance of considering both GHG emissions and sinks in evaluating the CFP of an agricultural system accurately [21].

Grazing systems are important resources in ruminant feeding, especially in areas where natural grasslands are part of the landscape. In the Mediterranean area, the livelihood of pastoral and agropastoral people depends largely upon rangelands, which are the major food source for their animals [22]. The Mediterranean agropastoral system [23] has evolved over time without the need for animal housing and feed supplies, but this practice has been increasingly lost due to the intensification of production. Nevertheless, because of their rusticity traits, in the Mediterranean area some native cattle breeds are still raised under this extensive pasture-based management system. 

While there are several CFP studies that have investigated intensive and semi-intensive cosmopolitan beef cattle systems, less attention has been dedicated to GHG emissions coming from pasture-based farms rearing native breeds.

Thus, by including the DNDC model in an assessment of soil GHG emissions and sinks, this study aimed to (i) perform a CFP study of pasture-based native beef cattle reared in a Mediterranean agropastoral system and (ii) assess the impact on a CFP of different agronomic practices such as tillage, fertilizing, and grazing management under current and future CC scenarios.

## 2. Materials and Methods 

### 2.1. Functional Unit and System Boundaries

**Figure 1:**
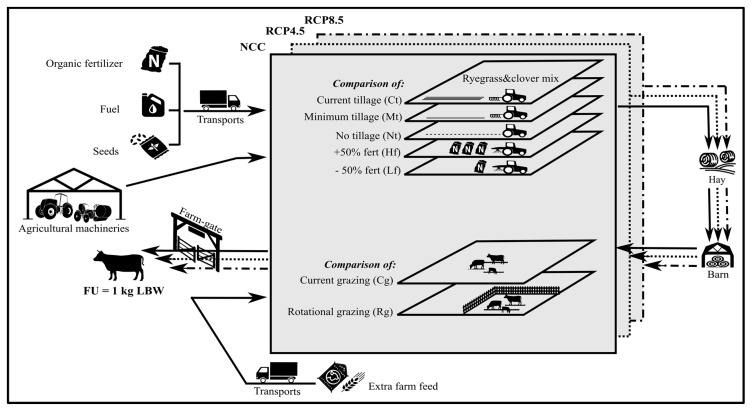
The system boundaries, goods, farm management practices, and climatic scenarios considered. NCC = current climate. RCP = representative concentration pathway. FU = functional unit. LBW = live body weight.

The functional unit (FU) chosen for the study was 1 kg of live body weight (LBW) of Maremmana beef cattle reared in a pasture all year round. Cradle-to-farm-gate system boundaries include all of the upstream processes of cattle beef farming until the animals leave the farm gate (Figure 1). Therefore, this study considered both direct GHG emissions coming from on-farm production processes (enteric fermentation, soil emissions, and fuel combustion) and the indirect GHG impacts related to the production and transport of auxiliary goods (seeds, organic fertilizers, extra farm feed, and fuel). GHG emissions deriving from the manufacturing of equipment (barns and sheds) as well agricultural machinery (tractors) were included. The following GHG sources were excluded from the boundaries of the system considered: the construction of fencing systems, the production of veterinary drugs, and animal respiration.

### 2.2. The Beef Cattle Pasture-Based Farming System

The beef cattle farm under study was located on the west coast of Central Italy within the Castelporziano natural reserve (41°42′50′’ N–12°24′03′’ E), which also hosts one of the three Italian presidential estates. The area, with an elevation ranging from 25 to 70 m above sea level, is about 6000 ha wide and is characterized by an inferior Mediterranean thermotype climate. Different land uses coexist within the natural reserve: Mediterranean lowland mixed forests, hydrophilous retro-dune wetland zones, Mediterranean scrub, grazed meadows, cultivated fields, and anthropic environments [24].

Within the beef cattle farm, which extends for about 480 ha inside the natural reserve, 280 livestock units (LUs) of native Maremmana beef cattle are currently organically reared in accordance with a year-round continuous unmanaged grazing management approach (hereinafter, Cg). The extensive grassland-based system described in this study can be considered to represent a typical farming system for Maremmana beef cattle reared in Central Italy [25].

The Maremmana breed is mainly spread throughout the Lazio and Tuscany regions, and it is an extraordinarily robust breed. Indeed, even though their slaughtering ages could be considered to be quite high (about 26–28 months old) compared to other Italian beef breeds such as Chianina, Marchigiana, and Romagnola (from 19 to 24 months old) [26], these animals are accustomed to living outdoors and are capable of surviving in harsh climates in which other breeds cannot adapt. After being weaned (6 months), calves are separated from cows and raised out to pasture. 

As a part of pasture integration, the animals annually consume (average of 2016–2018) about 815 t of dry matter (DM) ryegrass–clover hay mix produced on-farm, which amounts to about 8 kg of DM LU^−1^ day^−1^ (Table 1). During the two months preceding slaughter (fattening phase), the animals are finished with hay and concentrate mixes (Table 1) composed of (on a wet basis) 15% crude protein, 3.6% crude oils and fats, 9.7% crude fiber, 11% crude hash, and 0.4% sodium.

As far as agronomic practices, current tillage (Ct) includes presowing ploughing (30 cm), while current fertilization (Cf) includes the use of an organic compost fertilization (50 kg N ha^−1^ yr^−1^) (hereinafter, CtCf) (Table 1).

### 2.3. Alternative Farm Management Practices and Climate Scenarios 

In order to assess the potential impact of different agronomic practices on GHG emissions, two tillage alternatives (minimum tillage (Mt), including presowing ploughing (10 cm) coupled with Cf application rates (hereinafter, MtCf), and no-tillage (Nt) coupled with Cf application rates (hereinafter, NtCf)) were modelled as alternatives to the current CtCf. To assess the effects of organic fertilization on GHGs with respect to the organic fertilizer amount currently spread under Cf, a 50% increase (Hf) as well as a 50% decrease (Lf) were modeled for each tillage practice (Ct, Mt, and Nt).

Compared to fuel consumption under CtCf, a reduction of 13% and of 39% for MtCf and NtCf, respectively, was considered [27].

Furthermore, because on-farm hay production was affected when modeling the above alternative soil management strategies, resulting in hay yield deficits (CtLf), a certain amount of extra farm hay was assumed, and the related GHG emissions arising from its production and transportation (assuming 100 km of distance) were accounted for. On the other hand, when a yield surplus occurred, the hay was assumed to be sold and an economic allocation between cattle LBW and hay leaving the farm was adopted.

As for grazing management, by considering the annual growth rate [28] and yield (2 t of DM ha^−1^ yr^−1^) [29] of the grasslands, rotational grazing (hereinafter, Rg) was assumed to replace the current Cg. 

Of the entire grazing area (220 ha), six paddocks (~35 ha each) were assumed to be grazed twice from March to June (~10 days per turn and 45 days between turns), with a stocking rate of about 8.5 LU ha^−1^, while from July to February the whole area was assumed to be continuously grazed (1.3 LU ha^−1^).

Finally, in order to assess the interactions between current and alternative farm management practices and future climatic change, all of the management methods mentioned were modeled under three different long-term (from 2019 to 2089) climate pathways. 

For the first one (using in loco weather station climate data series (2009–2018)), no climate change was assumed to occur during the 70-year time frame considered (NCC). The second climate path reproduced Representative Concentration Pathway 4.5 (RCP4.5), which is based on the Fifth Assessment Report [30] of the Intergovernmental Panel on Climate Change (IPCC); while the third one was based on the less conservative RCP8.5 pathway. 

### 2.4. Life Cycle Inventory

The denitrification–decomposition (DNDC) model is made up of different submodels: (i) a soil climate submodel that simulates soil temperature moisture and Eh (redox potential); (ii) a plant growth submodel that estimates crop growth and its effects on soil (e.g., temperature, moisture, and available N); and (iii) a decomposition submodel that mainly simulates SOC and nitrogen dynamics. Depending on the derived soil environmental factors coming from the three upper-layer submodels, the denitrification or nitrification submodel is activated to simulate nitric oxide (NO) and nitrous oxide (N_2_O) gaseous emissions and nitrate $\left({\mathrm{NO}}_{3}^{-}\right)$ leaching. Moreover, the ammonium/ammonia (NH_3_) equilibrium is included in the nitrification model to estimate NH_3_ volatilization. The fermentation submodel calculates the release of methane (CH_4_) according to fermentation equations [31]. The DNDC adopts biogeochemical and empirical equations to simulate carbon and nitrogen biogeochemical cycles, including soil trace gas emissions [13].

The DNDC was used in this study to estimate direct and indirect N_2_O soil emissions, CH_4_ soil emissions, SOC dynamics, and forage crop growth. Site-specific DNDC data input on crop parameters, management activities, climate, and soil properties are reported in Table S1, Table S2, Table S3.

Although the DNDC has been run with a spin-up as low as two years [32], a 20-year spin-up is recommended to assure that different SOC pools reach equilibrium [33]. Thus, in order to reduce the uncertainties related to the initial model setting, in this study a 30-year spin-up was adopted. Specifically, the 2009–2018 climatic data (daily maximum and minimum air temperatures and precipitation) were repeated during the spin-up of all scenarios assessed. However, while a 10-year sequence was repeated randomly over 70 years within the NCC scenario model, climate projections were instead used within the RCP4.5 and RCP8.5 scenarios. In particular, Consortium for Small-scale Modelling and the Climate Limited-area Modelling community (COSMO-CLM) climate projections [34] were used by the Climate Model of the Euro-Mediterranean Center on Climate Change CMCC-CM [35] with the RCP4.5 and RCP8.5 emissions scenarios, and then the results were used in the DNDC model for the next 70 years modeled. 

Direct and indirect N_2_O emissions, as well as CH_4_ and soil carbon sources/sinks, were considered within each combination of climate scenarios per farm management policy considered. Particularly, the 70 years following the spin-up were selected for an analysis, with each combination assessed. 

The annual mean of the DNDC modeled N fluxes (leached and volatilized) were converted into N_2_O using the IPCC emissions factors [8]. The cumulative annual mean direct and indirect N_2_O emissions, as well as soil methane emissions, were converted into carbon dioxide equivalents (CO_2eqs_) using the IPCC 100-year global warming characterization factors of 28 for CH_4_ and 265 for N_2_O [30]. The average annual changes in the SOC (0–50 cm) were converted into CO_2eqs_ considering the atomic weight of C and the molecular weight of CO_2_, therefore multiplying the amount of the SOC source/sink by 3.67.

The enteric CH_4_ emissions were estimated using IPCC Tier 2 methodology [36] based on daily gross energy intake (GEI), the feed digestibility as a percentage of GEI (*DE%*), and the fraction of GEI converted to CH_4_ (*Y_m_*) (Table S4). Daily GEI was calculated considering LBW and the net energy required for maintenance (*NE_m_*), activity (*NE_a_*), lactation (*NE_l_*), pregnancy (*NE_p_*), and growth (*NE_g_*) of the different animal categories (e.g., cows, bulls, heifers, and fattened beef) (Table S4). The Y_m_ value adopted was 6.5% for grazing animals and 4.5% for those on fattening.

The environmental burdens associated with the production of fertilizers, fuel, packaging, seeds, extra farm feed, and durable goods (tractors and barns and sheds for the equipment) were considered (Figure 1). Because no electricity is involved in beef cattle rearing, fuel was the only input associated with energy consumption in the system. The transportation needed to support beef production was included, and for this reason, 3.5–7.5-t or 7.5–16-t lorries were considered.

The emission factors (EFs) adopted in this study (Table 2) were obtained from the Ecoinvent v3 database [37] and from the literature. Detailed farm input data are listed in Table S5 of the supplementary materials.

### 2.5. Statistical Analyses

The one-way analysis of variance (ANOVA) approach was adopted considering the tillage (Ct, Mt, and Nt), fertilization (Cf, Hf, Lf), grazing (Cg and Rg), and climate scenarios (NCC, RCP4.5, and RCP8.5) as fixed factors. Within each climate scenario, different combinations of tillage, fertilization, and grazing systems were modeled. The dependent variables were direct N_2_O, SOC, forage crop yield, and total CFP. The differences were tested using Tukey’s statistic and were considered significant for *p* < 0.05. Significant differences reaching a threshold lower than 5% (e.g., 0.01 or 0.001) are also highlighted in the text and in the figures according to conventional rules. All statistical tests were performed using Statistica^®^ 10 (Statsoft, Inc., Tulsa, OK, USA).

## 3. Results

The soil GHG emissions included within the total CFP values reported in this study were direct N_2_O fluxes, indirect N_2_O fluxes (the ${\;\mathrm{NO}}_{3}^{-}$ leached and NO and NH_3_ volatilized), net CH_4_ soil emissions, and SOC dynamics. However, because of the low incidence of net soil CH_4_ (almost null) and indirect N_2_O emissions (~3%) in total soil emissions, only the main sources of soil GHGs (direct soil N_2_O fluxes) and sinks (SOC dynamics) are discussed on the next paragraphs. The uncertainty values reported alongside the averages represent the standard error (*±se*).

### 3.1. Impact of Management and Climate on Direct Soil N_2_O Fluxes

**Figure 2:**
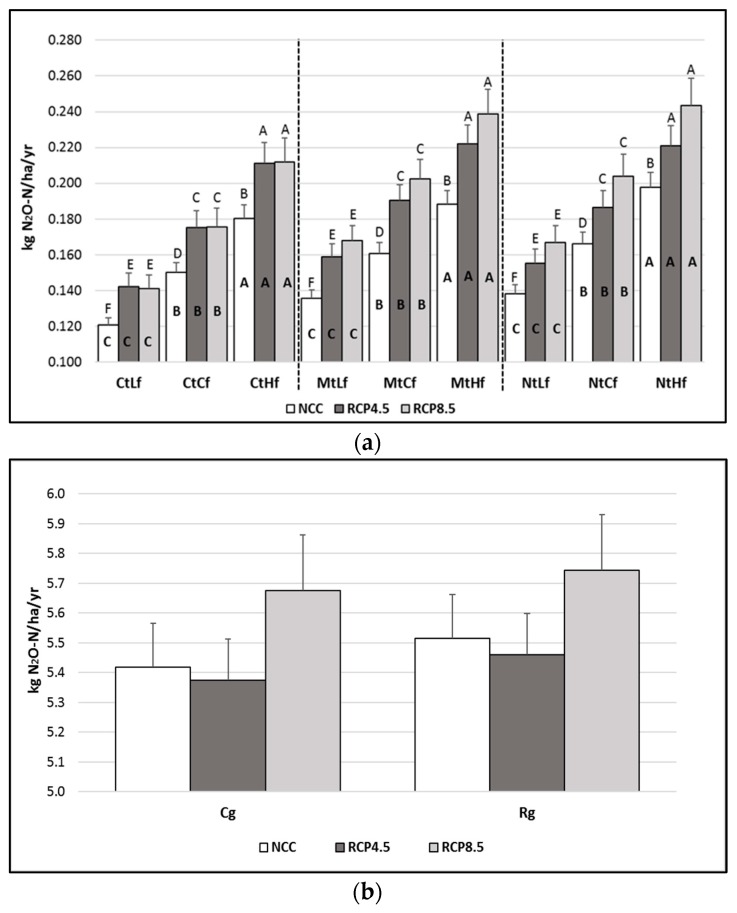
Denitrification–decomposition (DNDC)-modeled mean annual soil direct N_2_O fluxes (*±se*) from 2019 to 2089, as a result of different (**a**) farm management policies under diverse climate scenarios and different (**b**) grazing systems under diverse climate scenarios. Cf = current fertilization (50 kg N ha^−1^ yr^−1^); Cg = continuous grazing; Ct = current tillage; Hf = higher fertilization rate (+50% Cf); Lf = lower fertilization rate (−50% Cf); Rg = rotational grazing; NCC = current climate; RCP4.5 and RCP8.5 = Representative Concentration Pathways 4.5 and 8.5. The letters above the bars indicate differences between climate scenarios within each tillage per fertilization rate combination. The letters in bold inside the bars indicate differences between fertilization rates applied within each tillage per climate scenario combination. Different uppercase letters indicate statistical differences from Tukey’s test (*p* < 0.001). Different lowercase letters indicate statistical differences from Tukey’s test (*p* < 0.05).

The DNDC outputs showed a significant (*p* < 0.001) dose–effect relationship between direct N_2_O emissions and the organic fertilization rates applied (Figure 2a), while the tillage system results were not significant. 

Independently from the tillage and fertilization rate adopted, future climate RCP4.5 and RCP8.5 scenarios showed higher N_2_O emissions than did the ones occurring within the NCC (*p* < 0.001). As an example, the N_2_O emissions arising from the CtCf increased by about 15% (from 0.150 to 0.175 kg N_2_O-N ha^−1^ yr^−1^) when the RCP4.5 and RCP8.5 scenarios were modeled (Figure 2a). The N_2_O emissions differences between RCP4.5 and RCP8.5 were never significant.

No significant differences resulted between the two grazing management policies or between the different climate scenarios (Figure 2b).

### 3.2. Impact of Management and Climate on Soil Organic Carbon (SOC) Dynamics

**Figure 3:**
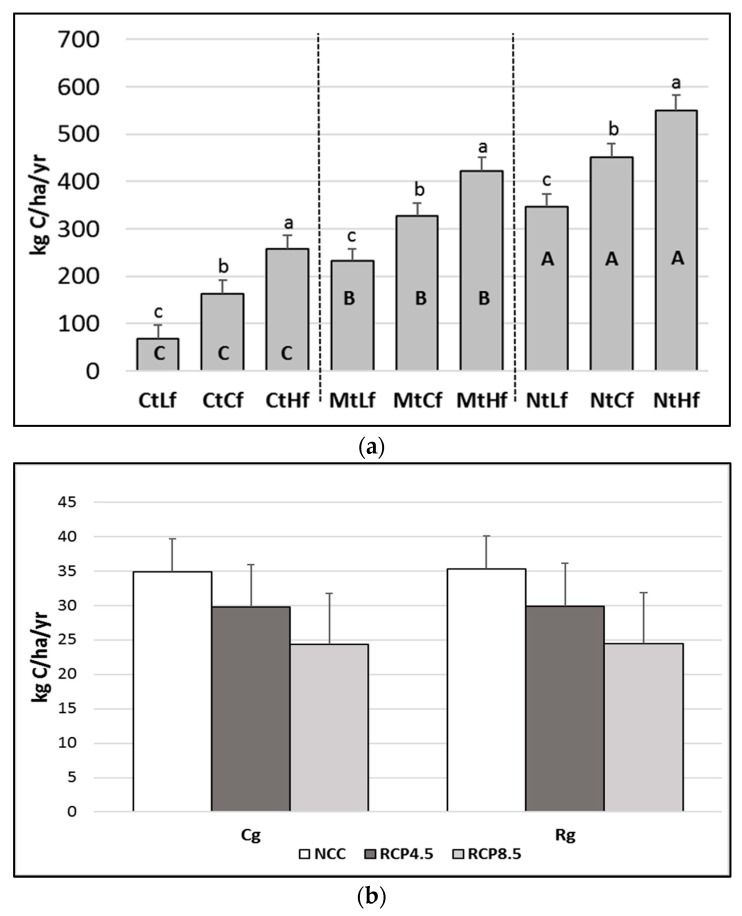
DNDC-modeled mean annual soil organic carbon (SOC) dynamics (0–50cm) (*±se*) from 2019 to 2089 as a result of different (**a**) farm management policies and (**b**) grazing systems under diverse climate scenarios. Cf = current fertilization (50 kg N ha^−1^ yr^−1^); Cg = continuous grazing; Ct = current tillage; Hf = higher fertilization rate (+50% Cf); Lf = lower fertilization rate (−50% Cf); Rg = rotational grazing; NCC = current climate; RCP4.5 and RCP8.5 = Representative Concentration Pathways 4.5 and 8.5. The letters above the bars indicate differences between fertilization rates, applied within each tillage. The letters in bold inside the bars indicate differences between each tillage per fertilization rate combination. Different uppercase letters indicate statistical differences from Tukey’s test (*p* < 0.001). Different lowercase letters indicate statistical differences from Tukey’s test (*p* < 0.05).

Figure 3a shows the SOC sink effect (0–50 cm) from the interactions of each tillage system per fertilization rate tested. The fertilization rates showed a significant (*p* < 0.05) dose-dependent effect on the SOC content, where Lf was the lowest and Hf was the highest within each tillage modeled. 

The SOC sinks estimated under Mt were higher than those modeled under Ct and lower than the ones simulated under Nt (*p* < 0.001).

The combination NtHf had the greatest effect on SOC sinks. The annual SOC sink amounts ranged from CtLf (the lowest value; 65 ± 21 kg C ha^−1^ yr^−1^) to NtHf (the highest value; 527 ± 23 kg C ha^−1^ yr^−1^).

The climate scenarios (NCC, RCP4.5, and RCP8.5) did not show significant effects on SOC sink rates within the farm management combinations modeled (result not shown).

No significant differences were observed between the two grazing management policies tested or between the different climate scenarios (Figure 3b).

### 3.3. Impact of Management and Climate on the Carbon Footprint of the Pasture-Based Cattle Beef System

Considering the current climate scenario (NCC), tillage system (Ct), fertilization rate (Cf), and grazing management system (Cg), the overall carbon footprint associated with the production of native Maremmana beef cattle (evaluated at the farm gate) was estimated to be 26.9 ± 0.7 kg CO_2eq_ kg LBW^−1^. With a 53% incidence, enteric fermentation was the main hot spot, followed by net soil GHG emissions (29%), fuel consumption (8%), transportation (6%), and extra farm feed (2%). The GHG emissions associated with seed production (1.3%), tractors (0.5%), and barns and sheds (0.2%) had only a marginal role (data not shown).

**Figure 4:**
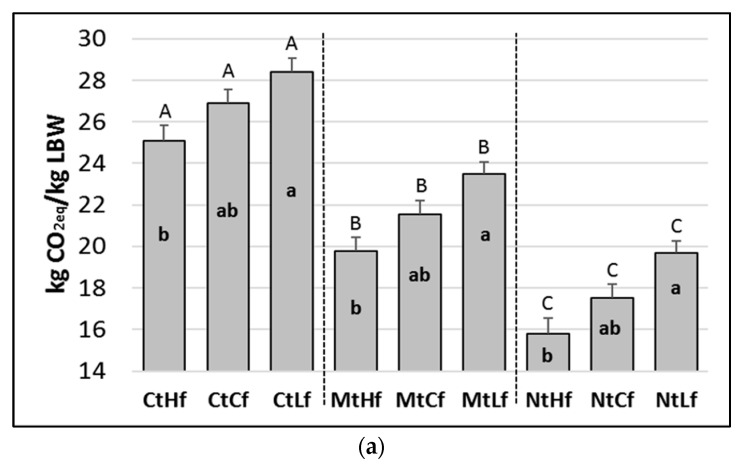

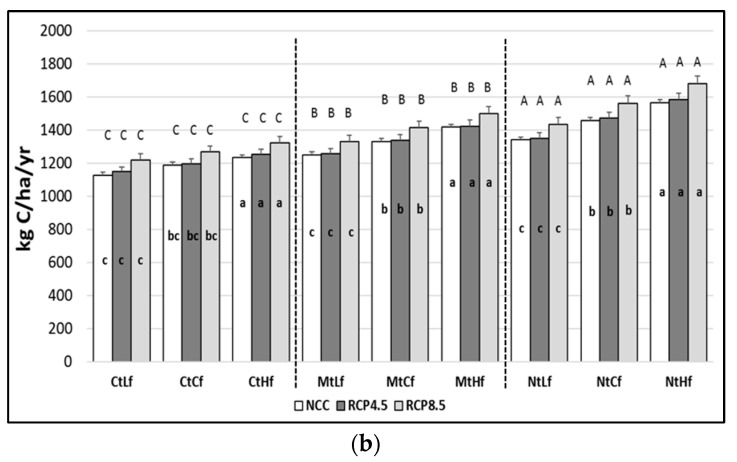
Mean annual (from 2019 to 2089) (**a**) overall carbon footprint (*±se*) as a result of different tillage types and fertilization rates and (**b**) crop yield (*±se*) as a result of different tillage types, fertilization rates, and climate scenarios. Cf = current fertilization (50 kg N ha^−1^ yr^−1^); Ct = current tillage; Hf = higher fertilization rate (+50% Cf); Lf = lower fertilization rate (−50% Cf); NCC = current climate; RCP4.5 and RCP8.5 = Representative Concentration Pathways 4.5 and 8.5. In (**a**), the letters above the bars indicate differences between each tillage per fertilization rate combination. The letters in bold inside the bars indicate differences between fertilization rates, applied within each tillage. In (**b**), the letters above the bars indicate differences between tillage types for each fertilization rate per climate scenario combination. The letters in bold inside the bars indicate differences between fertilization rates, applied within each tillage per climate scenario combination. Different uppercase letters indicate statistical differences from Tukey’s test (*p* < 0.001). Different lowercase letters indicate statistical differences from Tukey’s test (*p* < 0.05).

The adoption of less-invasive soil tillage systems has the potential to significantly reduce the overall CFP of pasture-based beef. Indeed, without considering the results of the modeled forage crop yield surplus, switching from CtCf to MtCf or NtCf reduced the CFP by 14% and 26%, respectively (*p* < 0.001) (data not shown). The CFP reductions (from switching to MtCf or NtCf) were more marked (20% and 35%, respectively) when the related crop yield surpluses were taken into account (Figure 4a). The climate scenarios did not induce any significant differences on the CFP resulting from the farm management combinations modeled (result not shown). With regard to fertilization rates, significant differences impacting total CFP (*p* < 0.05) were modeled (the lowest and highest rates of fertilizer application within each tillage) (Figure 4a).

Compared to current tillage (Ct), less-invasive tillage practices had a positive effect (*p* < 0.001) on the modeled crop yield, which increased under Mt and even further under Nt. Significant differences in the crop yield (*p* < 0.05) also resulted from different fertilization rates (Figure 4b). The climate scenario RCP8.5 induced a significant increase (*p* < 0.001) in crop yield compared to the crop yields modeled under NCC and RCP4.5 (significance not shown in figure).

Overall, the NtHf management combination showed the lowest CFP value for the beef production system under investigation. In particular, considering the environmental benefits from crop yield surpluses, the GHG emissions arising from the NtHf combination (15.8 kg CO_2eq_ kg LBW^−1^) were about 40% lower than those computed under the CtCf (Figure 4a).

The modeling comparing the Cg to the Rg system did not highlight significant effects on the CFP (data not shown).

## 4. Discussion

### 4.1. N_2_O Emissions Arising from the System

As expected, the DNDC showed N_2_O emissions that were N-dose-dependent. Particularly, 0.12% of the N added as organic compost volatilized as N_2_O emissions. Although comparisons to other studies were difficult due to the several factors involved in soil N_2_O emissions (e.g., management history, climate condition, and soil type), the results obtained in this study fell within the 0.01%–0.37% range found by other studies evaluating compost application N fluxes [41,42,43].

Due to the highly variable effects that conservation tillage practices have on N_2_O emissions, there is a considerable debate concerning the role of these practices in climate change (CC) [44]. Indeed, studies have reported that N_2_O emissions increase [45] or decrease [46]. In this study, compared to conventional tillage, the modeled conservation tillage practices did not show significant improvements, which has also been found in other studies [47].

Limited information exists about the possible impacts of CC on soil N_2_O emissions. A recent study [48] found that CC could induce a slight decrease in overall N_2_O emissions under different land use scenarios in the Mediterranean area. On the contrary, an Australian study that used the DNDC model to evaluate the N_2_O fluxes of rain-fed agricultural systems under RCP4.5 and RCP8.5 climate projections [49] found an increase in N_2_O emissions ranging from 34% to 75% compared to those occurring within the current climate scenario. In line with those findings, in this study, the RCP4.5 and RCP8.5 climate projections increased N_2_O emissions (15%–21%, respectively) in comparison to those occurring under the NCC scenario. The reason for these increased trends could be attributable to the warmer soil temperature modeled in the RCP4.5 and RCP8.5 scenarios. Indeed, increased soil temperature, which the model predicted under CC conditions, is expected to stimulate microbial activity and nitrification and denitrification processes [50].

According to the DNDC outputs, the mean annual N_2_O emissions arising from the conventional grazing system, Cg, amounted to about 5.4 kg N_2_O-N ha^−1^, and no differences resulted from switching to the Rg management system. The complex interactions between short-term weather conditions (e.g., warming and precipitation), land management practices (e.g., N inputs and tillage operations), and soil properties (e.g., bulk density, clay content, and water retention) make N_2_O emissions highly variable both temporally and spatially [51]. As a matter of fact, N_2_O emissions from grazed pastures have been reported to range from null in arid and infertile regions to up to 38.5 kg N_2_O-N ha^−1^ yr^−1^ in peat soils [52,53]. A study carried out on clay soil in Ireland reported a range of 1.7–6.3 kg N_2_O-N ha^−1^ yr^−1^ on no-graze perennial ryegrass grasslands receiving no fertilizer, and the range increased to 4.4–34.4 kg N_2_O-N ha^−1^ yr^−1^ when fertilizer and grazing were taken into account [54]. 

The results obtained for the long-term timeframe modeled in this study allow us to state that the adoption of conservation tillage practices such as Mt and Nt did not induce significant soil N_2_O emissions differences compared to those occurring under Ct. The 70-year time period modeled using the RCP4.5 and RCP8.5 climate pathways showed that annual N_2_O emissions did not increase under these two RCP scenarios, and because soil properties and management practices were kept unchanged during the modeled time period, the resulting increasing trend could be totally attributed to CC conditions. 

### 4.2. Soil Organic Carbon (SOC) Dynamics

Although one meta-analysis study has found that there are some beneficial effects due to switching from conventional to minimum or no-tillage practices [55], another study has highlighted that there is no effect from switching [56]. In our study, according to the C sink rates (0–50 cm) obtained from the modeled tillage alternatives (Figure 3a), the switch from Ct to Nt increased the SOC by about 0.27 t C ha^−1^ yr^−1^. It has been reported that increases in annual carbon sequestration rates can range from 0.1 up to 1 t ha^−1^ after conversion from conventional to no-tillage [57,58,59].

Although the link between C input and SOC sequestration could be considered to be a measure of C sequestration efficiency [6,60], there is no general relationship between these two parameters. Indeed, while some studies have reported soil C saturation after long-term repeated C inputs [61,62], others have shown a linear or logarithmical SOC sequestration [63,64]. With the tillage alternatives modeled in this study, no soil C saturation was reached at the end of the 70 years following the spin-up time, and a linear increase in SOC content with greater C input was observed. This linear trend could be partially explained by the starting low organic matter content (0.0085 kgC/kg soil) of the topsoil (0–10 cm) investigated in this study. Indeed, it has been reported that soil with depleted SOC generally indicates a long-term linear relationship between C inputs and sequestration rates [62].

The model clearly suggested that a higher proportion of compost applied to soil can significantly increase SOC stocks, which may provide an important C source of net sink both under current and future CC scenarios. Specifically, of the total organic C annually applied as compost, about 27% of it was constantly sequestered within the cultivated soil. These findings were consistent with those found by other studies that have investigated the effects of organic amendments on SOC sequestration [6,60].

A survey of the recent literature highlighted that there is no clear general relationship between grazing management and C sequestration [65,66]. As a matter of fact, contrasting findings can be found from the literature, with studies showing positive [7], null [67], or even worse [68] responses in terms of SOC from rotational grazing. The results of the long-term modeling from the present study did not highlight significant SOC differences between Cg and Rg. Nevertheless, the reason behind this finding could be attributable to the fact that with both Cg and Rg, the total number of animals was kept equal, and thus the overall manure in the fields was the same under both management structures. Furthermore, the DNDC lacks the ability to model grass regrowth dynamics (i.e., higher yields), which could potentially be achieved by controlling the physiological state of the meadow under Rg management: this may have further contributed to reducing the differences between the two grazing management policies investigated. In this regard, it would be important to conduct further investigations into the effects different grazing management policies could have on carbon sink in the Mediterranean context.

### 4.3. The Carbon Footprint (CFP) of Pasture-Based Cattle Beef

Besides the types of production systems involved (organic vs conventional), the CFPs of beef products are also strictly dependent on the cattle breed, finishing age, and type of diet. Although all of these factors, coupled with SOC sequestration rates, make a comparison of our results to other studies challenging, some conclusions can be drawn.

Recent studies [20,69] investigating the CFPs of typical Italian organic beef cattle farms (Chianina breed) have found that the GHG emissions at the farm gate were in the range of 20.9–23.3 kg CO_2eq_ kg LBW^−1^, lower than those obtained for the beef cattle system in the present study (26.3 kg CO_2eq_ kg LBW^−1^) under current conditions. The lower CFP value obtained for the Chianina breed compared to the Maremmana breed (present study) might have been due to the different production efficiencies of these breeds. Indeed, Chianina cattle have a younger slaughter age (~22 months) and a greater weight at slaughtering (~700 kg LBW head^−1^) compared to the Maremmana breed (~27 months, ~585 kg LBW head^−1^).

Compared to both the modeled [70] and observed [58] findings of other studies that have investigated the effects of compost applications, our modeling suggests that the use of organic fertilizers could result in a win–win situation where there is an increase in both C storage and the crop yield, which in turn could reduce the GHG emissions arising from on-farm forage crop production. Indeed, the greater N_2_O emissions modeled were associated with higher rates of simulated fertilizer applications, a result that was totally offset by the effects of the increased carbon sequestration rates. Specifically, considering the cradle-to-grave life cycle of organic compost, the ~0.32 kg CO_2eq_ emitted per kg of compost (produced, transported, and spread) was counterbalanced by the ~0.49 kg CO_2eq_ stored as organic matter in soil (data not shown). 

The impact that reduced tillage practices have on crop yield is controversial. Indeed, some studies have reported crop yield increases when conventional tillage was reduced to a minimum or to no-tillage [71], while others have reported similar [72] or decreased [73] yields. In this study, the switch from Ct to Mt and Nt resulted in modeled crop yield increases of 12% and 23%, respectively. The modeled yield increased to within the lower part of the range reported for rainfed crops under dry climates (6%–41%) [74,75], which could have been attributable to both the greater soil water conservation and greater SOC concentrations modeled with the Mt and Nt tillage practices.

The DNDC model outputs did not highlight any significant differences between the crop yields occurring under the NCC and RCP4.5 climate scenarios, while greater yields (8%) were modeled under RCP8.5. The main reason behind this trend could be attributable to the higher CO_2_ concentration considered within the RCP8.5 (468 ppm) scenario compared to the RCP4.5 (448 ppm) [76] and NCC (412 ppm) scenarios [77].

The replacement of Cg with Rg management did not lead to a significant improvement in terms of GHG emissions. However, although the DNDC model is a powerful tool for estimating the aboveground net primary production, significant uncertainties still exist when it is used to quantify the variation of the grazing effects on grasslands [78]. Indeed, by affecting species composition, primary production, and root biomass, grazing could have an overall impact on standing biomass that is more direct and rapid than that exerted by management practices and climate change [79]. In this regard, the validation of the model using site-specific field observations (e.g., the daily grass growth rate) needs to be explored in future work. Furthermore, on the animal side, by increasing the proximity of bulls to cows, a switch from Cg to Rg could have positive consequences (e.g., reducing the intercalving period) that were not accounted for in this case study.

Finally, although the conditions on the assessed beef farm are representative of the typical weather, soil, and management practices of native cow–calf systems bred in the Mediterranean area, further investigations within this ecoregion are needed for a better understanding of the GHG mitigation potential that is achievable using pasture-based systems.

## 5. Conclusions

The process-based model DNDC was used to quantify and evaluate the effects that different agronomic practices, grazing management policies, and climate projections could have on the GHG emissions arising from a pasture-based native beef cattle farm in a Mediterranean agropastoral system. The adoption of conservation tillage, such as minimum and no-tillage practices, was shown to be effective in mitigating GHG emissions: once implemented, they could enhance the amount of C sequestered in the soil and increase the yield of forage crops. Although the modeled increases from organic fertilization adoption induced greater soil N_2_O emissions, these were totally offset by the consequently greater SOC sink rates associated with this agronomic practice. Long-term modeling using the RCP4.5 and RCP8.5 scenarios improved our understanding of the effects that climate change scenarios could have on both N_2_O emissions and crop yields. The results from the 70-year modeling indicated that N_2_O emissions could increase with both climate change pathways, while increases in forage yields were found only within the RCP8.5 scenario. On the one hand, the adoption of no-tillage practices coupled with a higher rate of organic fertilization showed a high beef carbon footprint reduction potential, but on the other hand, the modeled switch from a continuous to a rotational grazing system did not lead to significant GHG emissions differences per unit of product. Agricultural carbon footprint studies that use process-based modeling have been useful in the evaluation of the effectiveness of mitigation strategies that can be implemented in these systems (because they model crop growth, SOC dynamics, and soil GHG emissions). However, testing the prediction abilities of these models using site-specific field observations needs to be explored in future work.

## Figures and Tables

**Table 1:** Main herd, weather, and crop management data.

Animals	Unit	Data
Cows	*n*	146
Breeding heifers	*n*	13
Beef cattle	*n*	89
Calves	*n*	114
Bulls	*n*	5
Cow live weight	kg	655
Heifer and steer live weight	kg	350
Typical slaughter ages	months	27
Fertility rate	%	80
Replacement rate	%	10
Age at first calving	months	36
Fattening period	months	2
Hay at pasture	kg DM LU^−1^ day^−1^	8
Hay at fattening	kg DM LU^−1^ day^−1^	6
Concentrates at fattening	kg DM LU^−1^ day^−1^	7
Average animals slaughtered per year	*n*	74
Average weight of the slaughtered animals	kg LBW head^−1^	585
Stocking rate on free-range pasture	LU ha^−1^	1.3
Pasture area	ha	220
**Cropping (CtCf)**	**Unit**	**Data**
Yield	kg DM ha^−1^	3000
Amount of organic fertilizer spread	t ha^−1^	1.3
Fertilizer N content	%	4
Fertilizer organic C content	%	50
Planting	month	October
Harvesting	month	May
Cropping area	ha	260
**Weather**	**Unit**	**Data**
Mean max. temperature	°C	21.5
Mean min. temperature	°C	11.6
Mean annual precipitation	mm	945

Animals and cropping values represent the 2016–2018 mean. Weather values represent the 2009–2018 mean. *n* = number; DM = dry matter; LBW = live body weight; LU = livestock unit; CtCf = current tillage and current fertilization.

**Table 2:** Emissions factors (EFs) list.

Input	Unit	EF (kg CO_2eq_ Unit^−1^)	Data Source
Compost (4% N content)	1 kg	0.03	[38]
Fuel production	1 kg	0.51	[37]
Fuel combustion	1 kg	3.17	[37]
Ryegrass–clover seeds	1 kg	1.62	[37]
Extra farm feed (concentrate)	1 kg	0.6	[39]
Extra farm hay	1 kg	0.28	[40]
Packaging paper	1 kg	0.88	[37]
Low-density polyethylene (LDPE)	1 kg	2.98	[37]
Buildings (lifespan: 50 years)	1 mq	168.9	[37]
Tractor (lifespan: 7000 working hours)	1 kg	5.73	[37]
Transport (lorry 3.5–7.5 t)	1 tkm	0.52	[37]
Transport (lorry 7.5–16 t)	1 tkm	0.22	[37]

tkm = ton per km.

## Supplementary Materials

The following are available online at https://www.mdpi.com/2076-2615/10/3/415/s1, Table S1: Model input data for the baseline ryegrass-clover mix cultivation; Table S2: Model input data for the baseline perennial grass grazed; Table S3: Model soil input data; Table S4: Average herd data (2016–2018) used for the enteric methane estimation; Table S5: Life cycle inventory (LCI) data.
