# Supplementary material for: Carbon Footprint of Mediterranean Pasture-Based Native Beef: Effects of Agronomic Practices and Pasture Management under Different Climate Change Scenarios
Source: Animals (Basel). 2020 Mar 2;10(3):415. doi: 10.3390/ani10030415 (PMC7143649; doi:10.3390/ani10030415)
Supplement: Supplementary file 1 [file animals-10-00415-s001.pdf]

Supplementary Materials:

Table S1. Model input data for the baseline ryegrass-clover mix cultivation

Crop	Parameters	Baseline data
Ryegrass-clover mix	Crop type	Annual grass
	Fraction of leaves + stems left in field after harvest	0.1
	Max. aboveground biomass (kg C ha^{-1} yr^{-1})	3,600
	Thermal degree days for maturity	1,350
	Water demand (g water/g DM)	115
	N fixation index (crop N/N from soil)	1.5
	Optimum temperature (degree C)	21
	Tilling method	Deep ploughing (30cm)
	Tilling date (month-day)	9-15
	Manure amendment applied	Compost
	Manure amendment applying date (month-day)	3-15
	Compost solid C/N ratio	14
	Organic C (kg C/ha)	700
	Organic N (kg N/ha)	50
	Compost application method	Surface spread
	Number of cuts	1
	Cutting date (month-day)	5-15
	Cut part	Grain-leaf-stem
	Cut fraction	0.9
	Length of the cultivation	1 year

Table S2. Model input data for the baseline perennial grass grazed

Crop	Parameters	Data
Perennial grass pasture	Crop type	Perennial grass
	Max. aboveground biomass (kg C ha^{-1} yr^{-1})	1,224
	Thermal degree days for maturity	1,000
	Water demand (g water/g DM)	200
	N fixation index (crop N/N from soil)	1.5
	Optimum temperature (degree C)	21
	Number of grazing applications	1
	Grazing starting date (month-day)	1-12
	Grazing ending date (month-day)	12-31
	Grazing hours per day	8
	Grazing intensity (heads ha-1)	1.3
	Additional feed (kg C $head^{-1}$ day^{-1})	3.2
	Feed C/N	20
	Excreta handle	Deposit in field

Table S3. Model soil input data

Dedicated area	Parameters	Data
Ryegrass-clover mix	Top soil texture	Sandy-loam
	Bulk density (g cm^{-3})	1.3879
	Soil pH	4.9
	Field capacity (wfps)	0.32
	Wilting point (wfps)	0.15
	Clay fraction	0.09
	Hydro-conductivity (m hr^{-1})	0.1248
	Porosity (0-1)	0.435
	SOC at surface soil (0-10cm) (kg C/kg Soil)	0.0085
	Initial nitrate concentration at surface soil (mg N kg^{-1})	0.5
	Initial ammonium concentration at surface soil (mg N kg^{-1})	0.05
Perennial grass pasture	Top soil texture	Clay-loam
	Bulk density (g cm^{-3})	1.1554
	Soil pH	8.2
	Field capacity (wfps)	0.57
	Wilting point (wfps)	0.27
	Clay fraction	0.41
	Hydro-conductivity (m hr^{-1})	0.015
	Porosity (0-1)	0.476
	SOC at surface soil (0-10cm) (kg C/kg Soil)	0.0214
	Initial nitrate concentration at surface soil (mg N kg^{-1})	0.5
	Initial ammonium concentration at surface soil (mg N kg^{-1})	0.05

Table S4. Average herd data (2016-2018) used for the enteric methane estimation

Parameters	Data
Lactating cows (heads yr^{-1})	146
Heifers and steers on pasture (heads yr^{-1})	142
Heifers and steers on fattening (heads yr^{-1})	12
Lactating cows (NEm) (MJ day^{-1})	41.7
Heifers and steers on pasture (NEm) (MJ day^{-1})	26.1
Heifers and steers on fattening (NEm) (MJ day^{-1})	36.6
Herds (NEa) (MJ day^{-1})	15
Lactating cows (NEl) (MJ day^{-1})	8.5
Pregnant cows (NEp) (MJ day^{-1})	2.9
Heifers and steers on pasture (NEg) (MJ day^{-1})	8.7
Heifers and steers on fattening (NEg) (MJ day^{-1})	18.2
Digestible energy (DE) expressed as % of gross energy for the fattening animals	75
Digestible energy (DE) expressed as % of gross energy for the rest of the herd	65
Ratio of net energy available in a diet for maintenance to digestible energy consumed (REM)	0.514
Ratio of net energy available for growth in a diet to digestible energy consumed (REG)	0.308

Table S5. Life cycle inventory (LCI) data

Input	Types		Amount year^{-1}
Auxiliary products	Organic fertilizer		312 t
	Packaging org. Fertilizer (LDPE)		624 kg
	Fuel		23,907 kg
	Ryegrass-clover seeds		9,600 kg
	Packaging of ryegrass-clover seeds (paper)		38 kg
	Extra farm feed		30,100 kg
	Packaging of extra farm feed (paper)		181 kg
**Input**	**Products**	**Types of transport**	**Distance (one way)**
Transports	Organic fertilizer	Lorry 3.5-7.5t	424 km
	Fuel	Lorry 7.5-16t	75 km
	Ryegrass-clover seeds	Lorry 3.5-7.5t	212 km
	Extra farm feed	Lorry 3.5-7.5t	321 km
	Waste	Lorry 3.5-7.5t	50 km
**Input**	**Types**	**Nr.**	**Area**
Agricultural buildings	Barns	2	300 mq
	Shed	1	150 mq
Agricultural machinery	Tractor	5	
